# LncRNA ADAMTS9‐AS2 suppresses the proliferation of gastric cancer cells and the tumorigenicity of cancer stem cells through regulating SPOP

**DOI:** 10.1111/jcmm.15161

**Published:** 2020-03-11

**Authors:** Feiran Wang, Chong Tang, Dong Xu, Yijie Tang, Yasu Jiang, Xuesong Gao, Junfei Xu

**Affiliations:** ^1^ Department of General Surgery Affiliated Hospital of Nantong University Nantong China; ^2^ Department of General Surgery Affiliated Hospital 2 of Nantong University Nantong China; ^3^ Department of General Surgery Huai’an First People's Hospital Huai'an Jiangsu China; ^4^ Medical College of Nantong University Nantong China

**Keywords:** ADAMTS9‐AS2, cancer stem cells, gastric cancer, Speckle‐type POZ protein, tumorsphere

## Abstract

Nowadays, research on CSCs is still in an initial stage, and there are few studies reporting the successful isolation and identification of CSCs. In the present study, we attempted to isolate CSCs through cultivating the cell line MKN45 in defined serum‐free medium and study the expression of stem cell markers or related proteins (Oct3/4, Sox2, Nanog and CD44) in CSCs. Moreover, immunofluorescence staining was performed to validate the stem cell markers of spheroid body‐forming cells. Further experiments were used to evaluate the SPOP expression in tumorsphere cells. In addition, ADAMTS9‐AS2 is a lncRNA that contributes to the genesis and development of many cancers, including gastric cancer (GC). We found ADAMTS9‐AS2 functioned as an anti‐oncogene and positively correlated with the expression of SPOP in GC tissues by combining bioinformatics analyses. Furthermore, we reported that ADAMTS9‐AS2 regulated the expression of SPOP in GC cells and tumorsphere cells to inhibit GC progression. Together, our results demonstrated that SPOP and ADAMTS9‐AS2 can be potential targets for GC treatment.

## 1 INTRODUCTION

Gastric cancer (GC) is one of the most common groups of malignancies in the world. There are one million new cases worldwide each year, mainly in developing countries, especially in China. The incidence of GC is second in various cancers in China.^1^ As in recent decades in China for GC prevention and control efforts, the effect of treatment for GC has occurred great changes, but the survival rate remains poor.^2^ The specific mechanism of the occurrence and development of GC are still not entirely clear. In these years, many studies have confirmed that there is a small part of cells in the tumour tissue, and they can possess self‐renewal and multi‐directional differentiation potential and strongly associated with the malignant degree of the tumour, drug resistance and metastasis, which plays a crucial role in the development and progression of tumour.^3^ Also, the American Association for Cancer Research identified these cells as self‐renewal, relatively static, multidrug‐resistant and multipotent cells in cancer tissue, as well as cells with stronger tumorigenicity and in vivo metastasis, as compared with other cancer cell lines. So, these cells were deemed cancer stem cells (CSCs).^3^ Tumour stem cells can proliferate migrate and produce tumours in an appropriate microenvironment. Some reports have been exhibited that GC tissues also exist some cells with stem cell properties, which play a vital role in the pathogenesis and development of GC.^4^, ^5^, ^6^ Therefore, to explore the mechanism of gastric CSCs and further studies on their participation is the key process of gastric carcinogenesis.

Speckle‐type POZ protein (SPOP), an E3 ubiquitin ligase adaptor, is a TRAF domain and POZ‐containing nuclear speckle‐related protein, which forms Cul3‐based ubiquitin ligases. SPOP binds with Cul3 ubiquitin ligase and interacts with substrate proteins to constitute a ubiquitin ligase complex.^7^ It can form heteromeric species, SPOP‐like (SPOPL). The molecular rheostat formed by SPOP and SPOPL can fine‐tune the E3 ubiquitin ligase activity. Recent studies have shown that SPOP correlated with the mediation of the ubiquitin and subsequent oncogenic hormone receptor SRC‐3 proteolysis, which suggests that SPOP plays a specified role in the inhibition of tumour.^8^ Recent research analysis found that SPOP protein was frequently mutated in some tumours, such as endometrial cancer,^9^ lung cancer^10^ and prostate cancer.^11^ Our previous studies reported that SPOP also had a similar situation in colon cancer,^12^ but it was not very clear in GC.

Long non‐coding RNAs (lncRNAs) are a kind of non‐coding RNAs with a length of more than 200 nucleotides with little or no protein‐coding potential.^13^ Increasing evidence suggests that lncRNAs play an important role in the development of malignancies, GC included. Considering the important functions of lncRNAs, it is necessary to identify and explore new lncRNAs in GC. By our bioinformatics analysis of the key lncRNAs in GC, we noticed that a lncRNA ADAMTS9‐AS2 was identified as a newly described tumour suppressor gene.

In the current study, we studied the SPOP expression in gastric CSCs and the different expressions between them and the primary adherent cells. And we further studied the influences of SPOP and ADAMTS9‐AS2 on proliferation, apoptosis, and the cycle of GC cells and spheroid formation of tumorsphere cells, in an attempt to explore the potential effects of ADAMTS9‐AS2 and SPOP in the initiation and progression of GC.

## 2 MATERIALS AND METHODS

### 2.1 Gastric cancer cell lines and Sphere formation

MKN45 cells were cultured in 1640 medium, supplemented with 10% FBS, and plated at the density of 1 × 10^6^ cells per 75 cm^2^ flask. Then, we passaged them upon cell confluence. Spheroid bodies were derived by placing the parental cells into serum‐free 1640 culture medium (containing 1% N2 supplement (Invitrogen), 2% B27 supplement (Invitrogen), 1% antibiotic mixture (100 mg/mL streptomycin and 100 U/mL penicillin G, Gibco), 20 ng/mL FGF2 (Chemicon) and 100 ng/mL EGF (Chemicon). The parental cells were plated in 96‐well ultra‐low attachment plate (Corning) at 100 cells per well. After two weeks, the number of suspension spheres was counted under the microscope (Olympus) and spheroid body formation was calculated. When the primary spheroid body reached the size of approximately 200‐500 cells per spheroid body, the spheroid bodies were separated at a density of 1000 cells per ml and 100 single‐cell suspension (100 μL) was seeded in each well of 96‐well ultra‐low attachment plate (Corning) in the serum‐free medium. After 2 weeks, the spheroid body formation of the second‐generation spheroid body was observed.

### 2.2 Immunofluorescence staining for stem cell markers

Cells plated onto poly‐L‐lysine–coated glass coverslips were fixed with 4% paraformaldehyde and then washed with PBS. Cells were permeabilized with 0.1% Triton X‐100/PBS for 10 minutes. Subsequently, cells were incubated with primary antibodies: CD44 (dilution of 1:100, Abcam), Oct‐3/4 (dilution of 1:100, Abcam), Sox2 (dilution of 1:100, Abcam) or Nanog (dilution of 1:100, Abcam). Furthermore, cells were probed with fluorescein isothiocyanate or Rhodamine‐tagged secondary antibodies. Consequently, the fluorescence was photographed under an inverted microscope (Leica).

### 2.3 Western blot analysis

Total protein was extracted by lysis buffer containing protease inhibitors (Promega). Equal amounts of protein separated by 10% sulphate‐polyacrylamide gel electrophoresis and then transferred to a polyvinylidene fluoride (PVDF) membrane. After incubation with anti‐CD44 1:200, anti‐Oct3/4 1:200, anti‐Sox2 1:200, anti‐Nanog and anti‐SPOP antibody (Abcam) overnight at 4℃, membranes were washed three times in TBST for 5 minutes and subsequently incubated secondary antibody with conjugated IRDye800 (1:5000, Rockland) at room temperature for 2 hours. The bands were scanned using an Odyssey infrared imaging system (LI‐COR, Lincoln) and quantified.

### 2.4 Real‐time quantitative PCR

Total RNAs were extracted using TRIzol (Invitrogen). The cDNA Synthesis Kit (Takara) was used for the synthesis of cDNA according to the manufacturer's instructions. qPCR was performed on the Mastercycler Ep realplex (Eppendorf 2S) to detect the mRNA level. The GAPDH (mRNAs) or U6 (lncRNAs) served as an internal control. The relative expression level for each target gene was calculated by using a 2^−ΔΔCt^ relative quantification method. Primer sequences were as follows: Oct4 forward: 5′‐AACGACCATCTGCCGCT‐3′, reverse: 5′‐CGATACTGGTTCGCTTTCTCT‐3′; Sox2 forward: 5′‐GAAAAACGAGGGAAATGGG‐3′; reverse 5′‐GCTGTCATTTGCTGTGGGT‐3′; Nanog forward: 5′‐CCTCCTCCCATCCCTCATA‐3′, reverse: 5′‐TGATTAGGCTCCAACCATACTC‐3′; CD44 forward: 5′‐CATCCCAGACGAAGACAGTCC‐3′, reverse 5′‐TGATCAGCCATTCTGGAATTTG‐3′; GAPDH forward: 5′‐GGCATCCTGGGCTACACT‐3′, reverse: 5′‐CCACCACCCTGTTGCTGT‐3′; SPOP forward: 5′‐TGACCACCAGGTAGACAGCG‐3′, SPOP reverse: 5′‐CCCGTTTCCCCCAAGTTA‐3′; lncRNA ADAMTS9‐AS2 forward: 5′‐CAGAAGGGGCTTGGTTGG‐3′, ADAMTS9‐AS2 reverse: 5′‐TCGTGTTCCTACCCTATTTTGA‐3′.

### 2.5 In vivo tumorigenicity experiments

Male athymic nude mice (nu/nu), 6 to 8 weeks old, were obtained from Nantong University Animal Center and were housed under pathogen‐free conditions in the barrier animal facility. For experiments, an equal number (2 × 10^6^) of freshly dissociated cells were suspended in 200 μL PBS, the spheroid body‐forming cells were injected subcutaneously into the groin of the four groups mouse (6 mice per group) and the parental cells were injected subcutaneously into the same area of the other four groups mice. The mice were observed for tumour growth every 10 days over 8 weeks. The grafts were removed and photographed, and the volumes were calculated based on the following formula: length (mm) × width^2^ (mm^2^)/2. The Animal Care Committee of Nantong University reviewed and approved these experiments.

### 2.6 Cell transfection

The ADAMTS9‐AS2 overexpression plasmid, the specific small interference RNA (siRNA) targeting SPOP (SPOP‐si) and scrambled siRNA control (si‐NC) were purchased from GENEPHARM. ADAMTS9‐AS2 sequence was amplified and inserted into the pcDNA3.1(+) vector. The sequences of primers were as follows: ADAMTS9‐AS2 forward: 5′‐CGGGATCCAAACTTGACGTACACACGCAGTC‐3′, ADAMTS9‐AS2 reverse: 5′‐TTCTGTTTTTATAATGTACATTAAATTAAGCGAGCTCCC‐3′; SPOP‐si (siRNA1): GGGTTAGATGAAGAAAGCA; siRNA2: GGGAGAAGAAACCAAAGCT; siRNA3: GTGGATTTCATCAACTATC. After transfection with the Lipofectamine 3000 (Invitrogen), cells were harvested for further functional assays.

### 2.7 Flow cytometry analysis

Cell apoptosis analysis was measured using flow cytometry. Cells were seeded in 6‐well plates (1 × 10^5^ cells/well) and treated with double staining with 100 nmol/L Annexin V‐FITC/PI. After transfection of 72 hours, the cells were washed with PBS at 4°C and then discarded from the plates by digestion with trypsin. After removing the supernatant and washing the cells twice with PBS, cells were resuspended with 500 μL of Annexin V‐FITC binding buffer and incubated with 5 μL of Annexin V‐FITC solution and 5 μL of PI for 15 minutes in the dark. The distribution of cell apoptosis was detected to calculate the percentage of cell death based on flow cytometry with the FACScan flow cytometer (BD Biosciences).

Moreover, cells were harvested and trypsinized, resuspended in ethanol, and stored at 4°C for 15 minutes. Afterwards, cells were centrifuged and resuspended in 450 μL PBS and 50 μL RNase A at 37°C for 30 minutes. Propidium iodide (PI, 50 μg/mL) was then treated with cells, which were cultured for 30 minutes in the darkness at room temperature. Cell cycle analysis was achieved on the FACScan flow cytometer (BD Biosciences) using the ModFit LT software.

### 2.8 Colony formation assay

Cells were seeded into 6‐well culture plates in complete growth medium and cultured for two weeks. Cells in the plates were fixed with 100% methanol for 10 minutes and stained with 0.5% crystal violet for 20 minutes at room temperature, and the number of colonies (>50 cells) was counted under an optical microscope. The experiment was repeated at least three times.

### 2.9 Statistical analysis

SPSS 19.0 software package (SPSS Inc) and Excel 2010 were used for statistical analyses. Student's *t* test was evaluated to analyse any significant differences. *P* < .05 was considered to be statistically significant.

## 3 RESULTS

### 3.1 Cancer stem‐like properties of tumorsphere cells

**Figure 1:**
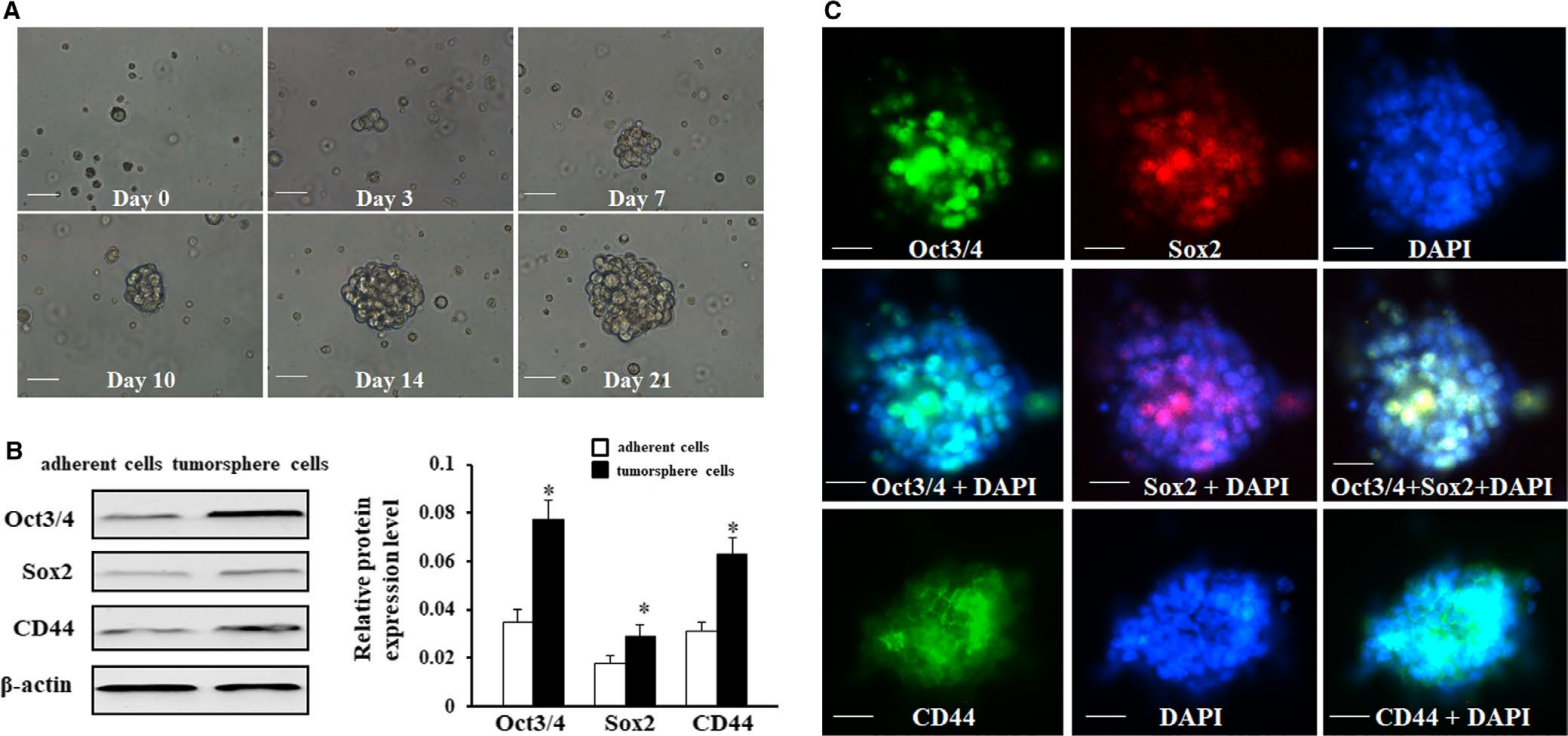
MKN‐45 cells formed the anchorage‐independent, self‐renewing spheroid bodies. A, The generation of a spheroid body from a single MKN‐45 cell was cultured in a 96‐well dish. Observation time‐point: days 0, 3, 7, 10, 14 and 21 and photographed under the light microscope (magnification × 200). B, Western blot analysis was used on adherent cells and tumorsphere cells, which indicated that the expression levels of Oct3/4, Sox2 and CD44 were dramatically more in tumorsphere cells than in adherent cells. C, Intracellular localization of Oct3/4, Sox2 and CD44 in tumorsphere cells by using immunofluorescence staining. And dual staining of Oct3/4 and Sox2 showed that Oct3/4‐positive stained cells were co‐stained with Sox2. DAPI was applied for the nuclear counterstain

To verify whether a cluster of gastric CSCs exists in GC cell lines, MKN45 cells were cultured in a serum‐free medium. The cells in a suspension state, with the extension of time, the spheroid cells gradually increased in size and number increase exponentially. Following 21 days of culture, the spheroid body‐forming cells were observed ranging between 50 and 200 cells per sphere. After the stem cell‐conditioned culture, these tumour cells from MKN45 cells that grew in three‐dimensional spheroid clusters were called tumorsphere cells. As shown in Figure 1A, the phase images showed the process of single MKN45 cells forming a tumour spheroid body. After isolation of tumorsphere cells in MKN45 cells, we detected the protein levels of stem cell markers (Oct3/4, Sox2 and CD44) to determine the stem cell characteristics in adherent cells and tumorsphere cells. The results of Western blot revealed that Oct3/4, Sox2 and CD44 were markedly up‐regulated in tumorsphere cells compared with adherent cells (Figure 1B). In addition, immunofluorescence staining was examined to evaluate the subcellular localization of Oct3/4, Sox2 and CD44 in tumorsphere cells. Double immunofluorescence staining showed that colocalization of Oct3/4 and Sox2 could be found in spheres, which localized to the nucleus of tumorsphere cells. Moreover, the staining of CD44 indicated that CD44 was positively stained in the membrane of tumorsphere cells by using immunofluorescence staining (Figure 1C).

**Figure 2:**
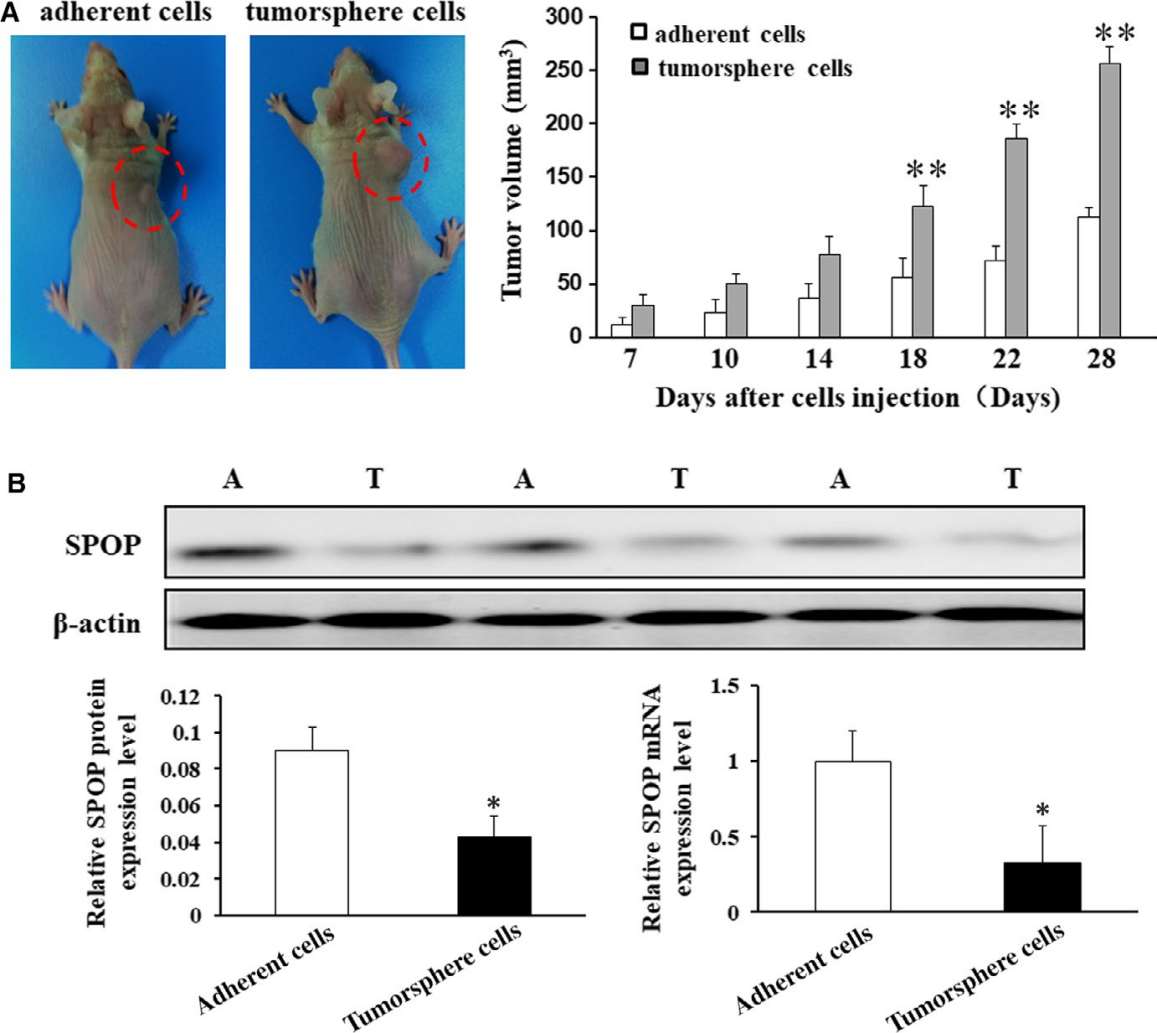
The expression of SPOP in tumorsphere cells and adherent cells. A, The experiment of tumour xenografts in nude mice in vivo showed that 2 × 10^4^ tumorsphere cells and 2 × 10^6^ adherent cells can both form a xenograft tumour after subcutaneous injection, but tumorsphere cells generated subcutaneous tumors with larger volume and quicker compared with those from adherent cells. B, Western blot and qPCR analyses were performed to detect the expression of SPOP in tumorsphere cells (T) and adherent cells (A). The experiments were repeated in triplicate. These above results indicated that the expression of SPOP in tumorsphere cells was significantly less than that in adherent cells. *P < .05, **P < .01

To confirm whether tumorsphere cells exhibited more tumorigenic than adherent cells in vivo, we examined the tumorigenic capacity between tumorsphere cells and adherent cells. Then, different numbers of tumorsphere cells and adherent cells were injected into the nude mice. We observed that tumorsphere cells formed subcutaneous tumour nodules with larger volume and quicker compared with those from adherent cells in injected mice (Figure 2A). Representative macroscopic appearances of subcutaneous xenografts in nude mice of tumorsphere cells and adherent cells were shown in Figure S1. These above results indicated that tumorsphere cells were more tumorigenic in vivo.

### 3.2 The expression of SPOP in gastric cancer stem cells

**Figure 3:**
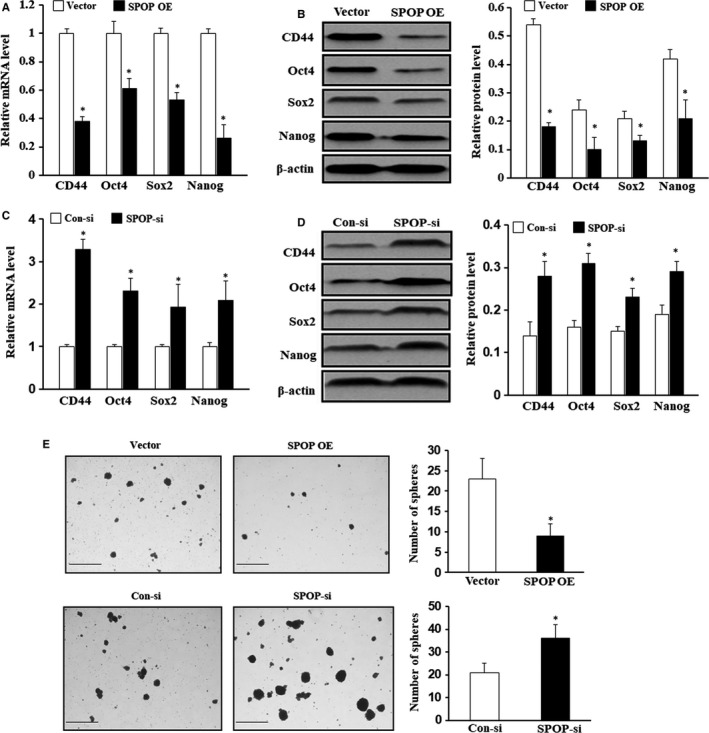
SPOP regulates stem‐like capacities of gastric CSC. A,B, The mRNA and protein expression levels of CSCs markers (CD44, Oct4, Sox2 and NANOG) were examined by qPCR and Western blot in tumorsphere cells SPOP overexpression plasmid and control vector cells. C,D, The mRNA and protein expression levels of CD44, Oct4, Sox2 and NANOG in tumorsphere cells transfected with SPOP siRNA or scramble siRNA were also examined by qPCR and Western blot analysis. E. A number of spheres per well were quantified on spheroid formation assay for tumorsphere cells transfected with SPOP overexpression plasmid or SPOP siRNA, respectively. *P < .05

Interestingly, one previous study showed that SPOP was greatly down‐regulated in GC tissues by using Western blot analysis.^14^ To examine the role of SPOP in gastric CSCs, we evaluated the expression level of SPOP by Western blot and qPCR analysis in tumorsphere cells and adherent cells. The above results indicated that SPOP expression was down‐regulated in tumorsphere cells (Figure 2B). We further investigated whether SPOP has an effect on the stem cell potential of tumorsphere cells. The transfection efficiency was confirmed by flow cytometry. Flow cytometric analysis confirmed that the transfection achieved high efficiency (Figure S2A). The expression levels of SPOP were examined by Western blot in MKN45 cells transfected with SPOP‐si and SPOP overexpression plasmid (Figure S2B and S2C). The results of qPCR and Western blot analysis demonstrated that tumorsphere cells transfected with SPOP overexpression plasmid exhibited significantly decreased expression of CD44, Oct3/4, Sox2 and NANOG compared with their control cells (Figure 3A and B, *P < .05). Whereas, knockdown of SPOP markedly promoted the mRNA and protein levels of CD44, Oct3/4, Sox2 and NANOG compared with the cells transfected with scramble siRNA (Figure 3C and D, *P < .05). Consistently, overexpression of SPOP in tumorsphere cells significantly formed fewer tumorspheres on spheroid formation assay, whereas, knockdown of SPOP dramatically enhanced tumorspheres (Figure 3E, *P < .05). Collectively, these results suggest that SPOP inhibits the cancer stem‐like capacity of tumorsphere cells.

### 3.3 Identification of ADAMTS9‐AS2 as a candidate lncRNA

A prediction model based on protein‐coding genes‐related lncRNAs was established by calculating the Pearson correlation coefficient as described in Supplemental Methods. The heatmap indicated the SPOP gene‐related lncRNAs between GC tissues and adjacent normal tissues, ADAMTS9‐AS2 included (Figure S3). Moreover, statistical software R and edgeR packages were performed to analyse the differentially expressed lncRNAs between tumour and normal tissues. The volcano plot was shown in Figure S4A. The top 100 differentially expressed lncRNAs in GC tissues were screened out and are presented in Figure S4B. As shown in the volcano plot and heatmap, ADAMTS9‐AS2 was a lowly expressed lncRNA in GC. Furthermore, a total of 375 tumour tissues and 32 normal tissues with ADAMTS9‐AS2 expression data from the TCGA database across all patient characteristics were analysed. We verified the expression level of ADAMTS9‐AS2 using LIMMA software in TCGA data and found that the down‐regulation of ADAMTS9‐AS2 was in tumour tissues compared with normal tissues (Figure S4C, *P* < .001). To further validate the results, we examined the expression of ADAMTS9‐AS2 and the relationship between ADAMTS9‐AS2 expression and the expression of SPOP in tumour tissues by using the GEPIA database (Figure S5).

### 3.4 ADAMTS9‐AS2 partly reversed the effects of SPOP‐mediated GC progression

**Figure 4:**
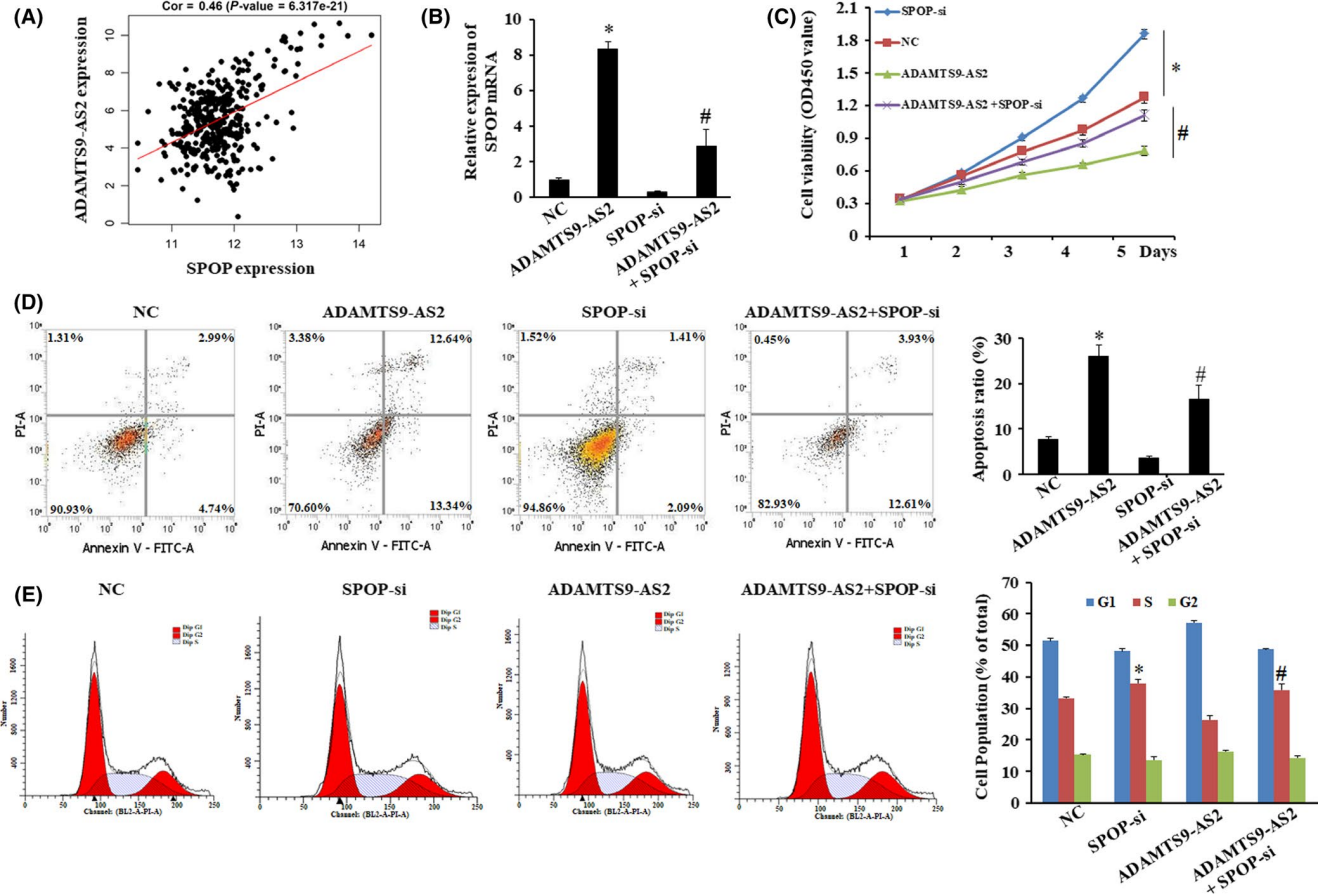
Effects of ADAMTS9‐AS2 and SPOP on proliferation, apoptosis, and the cycle of GC cells. A, Pearson's correlation analysis of the relationship between ADAMTS9‐AS2 and SPOP expression levels in GC tissues from STAD patients in the TCGA data by bioinformatics analysis. B, qPCR analysis exhibited that the expression levels of SPOP and ADAMTS9‐AS2. C, The proliferation of MKN45 cells in four groups was examined by MTT assay. D, Cell apoptosis was detected by Annexin V/PI with flow cytometry in MKN45 cells. The apoptotic evaluation was calculated by the percentage of apoptotic cell number in total cell number. E, Cell cycle detected by flow cytometry in MKN45 cells. Histogram represented the percentage of cells in G1, S and G2 cell cycle phases. *P < .05, compared with the NC group; ^#^ *P* < .05, compared with ADAMTS9‐AS2 group

ADAMTS9‐AS2 expression was positively correlated with the SPOP expression in GC tissues from Stomach adenocarcinoma (STAD) patients in the TCGA data by using the R package (Figure 4A). Based on the above‐described findings, we detected the effects of SPOP and ADAMTS9‐AS2 expression in GC cells by qPCR (Figure 4B). The expression levels of SPOP mRNA in MKN45 cells transfected with pCMV‐ADAMTS9‐AS2 were significantly elevated compared with the NC group (*P < .05). Additionally, ADAMTS9‐AS2 still markedly promoted the expression of SPOP in cells transfected with si‐SPOP (^#^ *P* < .05). Besides, the results of MTT assays indicated that ADAMTS9‐AS2 significantly inhibited cell proliferation while si‐SPOP dramatically promoted cell proliferation (Figure 4C, *P* < .05), but the cell viability is not significantly changed by MKN45 co‑transfected with ADAMTS9‐AS2 + si‐SPOP, in comparison with NC group.

Moreover, the results of the cell cycle showed si‐SPOP accelerated G1 to G2 phase, and ADAMTS9‐AS2 attenuated the G1‐phase arrest. While we also found an increase in the percentage of cells in the S phase in the ADAMTS9‐AS2 + si‐SPOP group compared with the NC group (Figure 4E, *P* < .05). Furthermore, flow cytometry analysis also indicated that the apoptosis rate of MKN45 cells was promoted by ADAMTS9‐AS2 and ADAMTS9‐AS2 with si‐SPOP compared with the NC group (Figure 4D, *P < .05).

### 3.5 ADAMTS9‐AS2 was involved in SPOP‐mediated proliferation and spheroid formation

**Figure 5:**
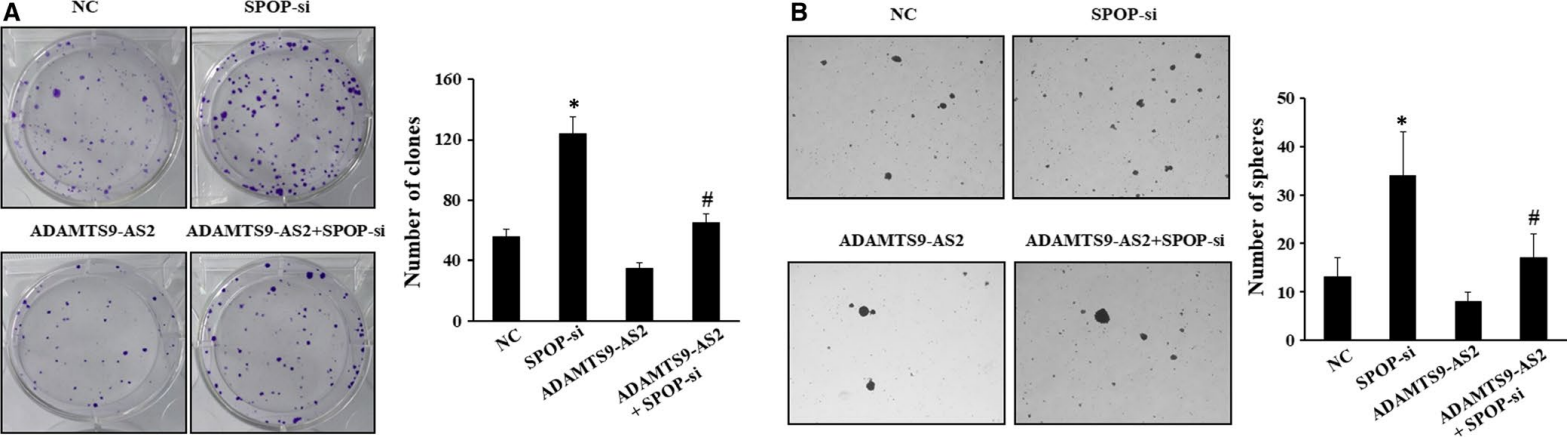
Effects of ADAMTS9‐AS2 and SPOP on colony formation of MKN45 cells and spheroid formation of tumorsphere cells. A, Colony formation assays were performed to detect the effects of growth in MKN45 cells transfected with SPOP siRNA or pCMV‐ADAMTS9‐AS2. The number of clones was analysed quantitatively. B, A number of spheres per well were quantified on spheroid formation assay for tumorsphere cells transfected with SPOP siRNA or pCMV‐ADAMTS9‐AS2. *P < .05, compared with the NC group; ^#^ *P* < .05, compared with ADAMTS9‐AS2 group

To further determine the effects of ADAMTS9‐AS2 and SPOP on GC cells and tumorsphere cells, colony formation assay and spheroid formation assay were applied, respectively. After being transfected with pCMV‐ADAMTS9‐AS2, the number of clones and spheroid formation were significantly decreased, while the cells transfected with si‐SPOP increased sharply. Besides, MKN45 cells and tumorsphere cells cotransfected with pCMV‐ADAMTS9‐AS2 or SPOP siRNA were relatively reduced compared with a si‐SPOP group (Figure 5A,B, *P* < .05). The above results demonstrated that ADAMTS9‐AS2 suppressed the proliferative ability of GC cells and spheroid formation of tumorsphere cells was mediated by the down‐regulation of SPOP in vitro.

## 4 DISCUSSION

Nowadays, in order to improve the understanding of the mechanisms leading to GC, new therapeutic strategies may be discovered and developed for GC therapies. Recent reports suggest that researchers have been absorbed in identifying and targeting CSCs. CSCs have become a popular topic in cancer research, which may be helpful to improve the overall understanding of the occurrence of GC and explain the treatment failure for GC. CSCs are a small subgroup of cells with stem cell properties, including a self‐renewal, tumour‐initiation, higher tumorigenicity, metastatic potential and chemotherapeutic resistance.^3^ In order to study the characteristics of CSCs, including the occurrence and recurrence of tumours, many types of research have investigated the methods of isolating CSCs from cancer cell lines. Recently, the anchorage‐independent tumorsphere culture of stem cells was contributed to investigate CSCs.^15^ And sphere formation was regarded as to be an effective approach of isolation or enrichment of stem cell‐like cells from multiple tumour types.^16^, ^17^ In this paper, in order to isolate gastric CSCs from MKN‐45 cells, the spheroid body formation assay was conducted. We found that, as time increased, the cell volume of the suspended spheres increased, and the number increased. Further studies showed that the tumorsphere cells from MKN45 cells possessed higher self‐renewal capacity and differentiation potential abilities than adherent cells.

Recently, the expression of CD44,^18^ Oct3/4^19^ and Sox2^20^ have been found in some CSC‐like cells, indicating that its expression may be involved in self‐renewal and tumorigenesis by activating downstream target genes. And these genes are pluripotent transcription factors and have been identified as stem cell markers. To further identify gastric CSCs, these above different cell‐specific surface markers have been assessed. In the present study, our results showed that the expression of Oct3/4, Sox2, CD44 and Nanog was up‐regulated in tumorsphere cells. These results indicated that our sorted tumorsphere cells appeared to be enriched with gastric CSCs. In this way, we also verified the existence of gastric CSCs, and we have applied the method^21^ to isolate CSCs by serum‐free cell culture. More interestingly, the tumorigenicity experiment also exhibited that the tumour formation rate of tumorsphere cells sorted from MKN‐45 cell lines was dramatically higher than that of adherent cells. Besides, SPOP is a 374‐amino acid protein that comprises a POZ domain downstream of the predicted TD, which has been previously positioned in the nucleus in speckled mode.^22^ SPOP is now considered a tumour suppressor because its expression has been shown to inhibit SRC‐3–mediated carcinogenic signalling and carcinogenesis.^8^ A recently published study showed that SPOP suppressed gastric cancer cell invasion and proliferation by regulating Hh/Gli2 signalling pathway.^14^ But so far, there is no research demonstrated that SPOP expression focuses on gastric CSCs. In this study, Western blot and qPCR assays indicated that the expression of SPOP was down‐regulated in tumorsphere cells; besides, spheroid formation assay showed that the tumorsphere‐SPOP cells formed fewer tumorspheres, indicating that SPOP suppressed the stemness of gastric CSCs. Furthermore, the number of tumorspheres on the tumorsphere‐SPOP group was dramatically smaller than that of the control vector group, which indicated that the overexpression of SPOP suppressed the growth of gastric CSCs in vitro.

By some previous studies, we noticed that lncRNA ADAMTS9‐AS2 may be strongly associated with some important cancer‐related genes.^23^ In this study, combining bioinformatics analyses, we discovered the down‐regulation of ADAMTS9‐AS2 and SPOP in GC cells and revealed that ADAMTS9‐AS2 could positively regulate the expression of SPOP. In recent years, emerging research has revealed that lncRNA ADAMTS9‐AS2 is identified as a new tumour suppressor gene and is the antisense transcript of ADAMTS9.^24^ LncRNA ADAMTS9‐AS2 participates in the development of various tumours. Previous researches have reported that down‐regulation of ADAMTS9‐AS2 was in glioma,^23^ colorectal cancer,^25^ ovarian cancer^26^ and clear cell renal cell carcinoma.^27^ In addition, other reports revealed that ADAMTS9‐AS2 inhibited the tumour progression by the miR‐223‐3p/TGFBR3 axis in lung cancer.^28^ Shi et al also found that ADAMTS9‐AS2 is lowly expressed in breast cancer tissues and drug‐resistant breast cancer cells. ADAMTS9‐AS2 inhibited PTEN expression and enhanced tamoxifen resistance through targeting microRNA‐130a‐5p.^29^ Moreover, one previous finding has indicated that overexpression of ADAMTS9‐AS2 may suppress the proliferation of GC cells, inhibit the migration and invasion of cancer cells and induce apoptosis. The activation of the PI3K/Akt pathway may be an important regulatory mechanism of ADAMTS9‐AS2 in GC.^24^ However, the role of ADAMTS9‐AS2 in gastric CSCs remains unclear. Moreover, in our study, we demonstrated that overexpression of ADAMTS9‐AS2 could up‐regulate the expression of SPOP, thus suppressing cell growth, proliferation, promote apoptosis and block cell cycle progression of GC and spheroid formation of tumorsphere cells, which suggested that ADAMTS9‐AS2 played a crucial role in inhibiting GC progression by targeting SPOP. Our results also suggested that ADAMTS9‐AS2 expression may be regulated by DNA methylation of methyltransferases DNMT1.^23^ Besides, the expression of SPOP is also controlled by promoter hypermethylation.^30^ Therefore, we hold the opinion that the process that lncRNA ADAMTS9‐AS2 was involved in SPOP expression regulation in GC might be regulated by DNA methylation. Further researches are needed to study the more precise mechanisms.

Taken together, this study demonstrated that tumorsphere cells from MKN45 cell lines possess gastric CSCs properties. It is apparent that stem cell markers Oct3/4, Sox2 and CD44 maintain the stemness of gastric cancer in tumorsphere cells. SPOP played a vital role in the forming process of CSCs. Moreover, ADAMTS9‐AS2 acted as a tumour suppressor that inhibited the progression of GC through regulating the SPOP, and these findings indicated that SPOP and ADAMTS9‐AS2 can be potential targets for GC treatment.

## CONFLICT OF INTEREST

The authors declare that they have no competing interests.

## AUTHORS CONTRIBUTIONS

Feiran Wang and Junfei Xu conceive research and design of the study. Chong Tang and Yasu Jiang analysed and interpreted the data. Dong Xu and Yijie Tang contributed to statistical analysis. Feiran Wang and Dong Xu drafted the manuscript. Chong Tang and Xuesong Gao critically reviewed the manuscript. All authors approved the final manuscript.

## Supporting information

Supplementary Material

Supplementary Material

## Data Availability

The data that support the findings of this study are available from the corresponding author upon reasonable request.
