# Supplementary material for: LncRNA ADAMTS9‐AS2 suppresses the proliferation of gastric cancer cells and the tumorigenicity of cancer stem cells through regulating SPOP
Source: J Cell Mol Med. 2020 Mar 11;24(8):4830–8. doi: 10.1111/jcmm.15161 (PMC7176847; doi:10.1111/jcmm.15161)
Supplement: Supplementary file 1 — Supplementary Material [file JCMM-24-4830-s002.docx]

**SUPPLEYMENTARY INFORMATION**

**SUPPLEYMENTARY METHODS**

**Analysis of expression of lncRNAs and protein-coding genes**

We examined the expression correlation between lncRNAs and protein-coding genes by calculating the Pearson correlation coefficient in the R package, which determined the co-expression relationships of the lncRNAs and protein-coding genes. The lncRNAs with |Pearson correlation coefficient| > 0.5 and P-value < 0.01 were considered to be protein-coding genes-related lncRNAs.[^1-3^](#_ENREF_14) We further identified the cluster of protein-coding genes-related lncRNAs by using the “pheatmap” package in R.

**Explore the differentially expressed lncRNAs**

The RNA sequencing (RNA‐Seq) data were derived from the The Cancer Genome Atlas (TCGA) database. There are 375 GC tumor tissues and 32 adjacent normal tissues with available lncRNASeq. We used the R and Bioconductor package of edgeR to explore the significantly differentially expressed lncRNAs between tumor and normal tissues. The cut‐off value was |log_2_FC| > 2 and FDR < 0.001 (FC, fold change; FDR, false discovery rate). We further identified the cluster of differentially expressed lncRNAs by using the “pheatmap” package in R.

**REFERENCES**

1. Guo Q, Cheng Y, Liang T, et al. Comprehensive analysis of lncRNA-mRNA co-expression patterns identifies immune-associated lncRNA biomarkers in ovarian cancer malignant progression. *Sci Rep*. 2015; 5: 17683.

2. Chen W, Zhang X, Li J, Huang S, Liu C. Comprehensive analysis of coding-lncRNA gene co-expression network uncovers conserved functional lncRNAs in zebrafish. *BMC Genomics.* 2018; 19: 112.

3. Alaei S, Sadeghi B, Najafi A, Masoudi-Nejad A. LncRNA and mRNA integration network reconstruction reveals novel key regulators in esophageal squamous-cell carcinoma. *Genomics.* 2019; 111:76-89.

**SUPPLEYMENTARY FIGURES**

**FIGURE S1**

**
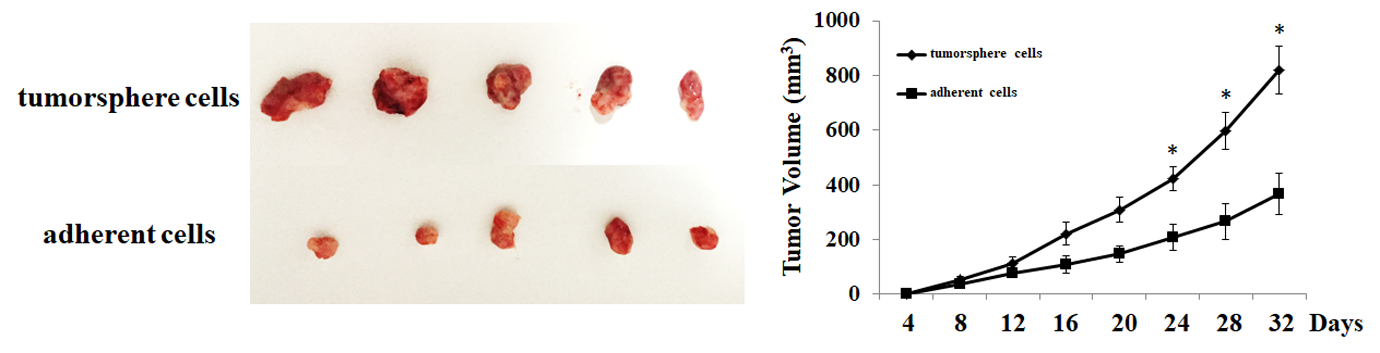
**

**FIGURE S1** Tumorsphere cells and adherent cells were injected subcutaneously into the right flank of nude mice. Representative macroscopic appearances of subcutaneous xenografts removed from bodies 32 days after implantation. The tumor volumes were measured every 4 days from day 4 after transplantation. There are significant differences in tumor size between the two groups (^*^*P* < 0.05)

**FIGURE S2**


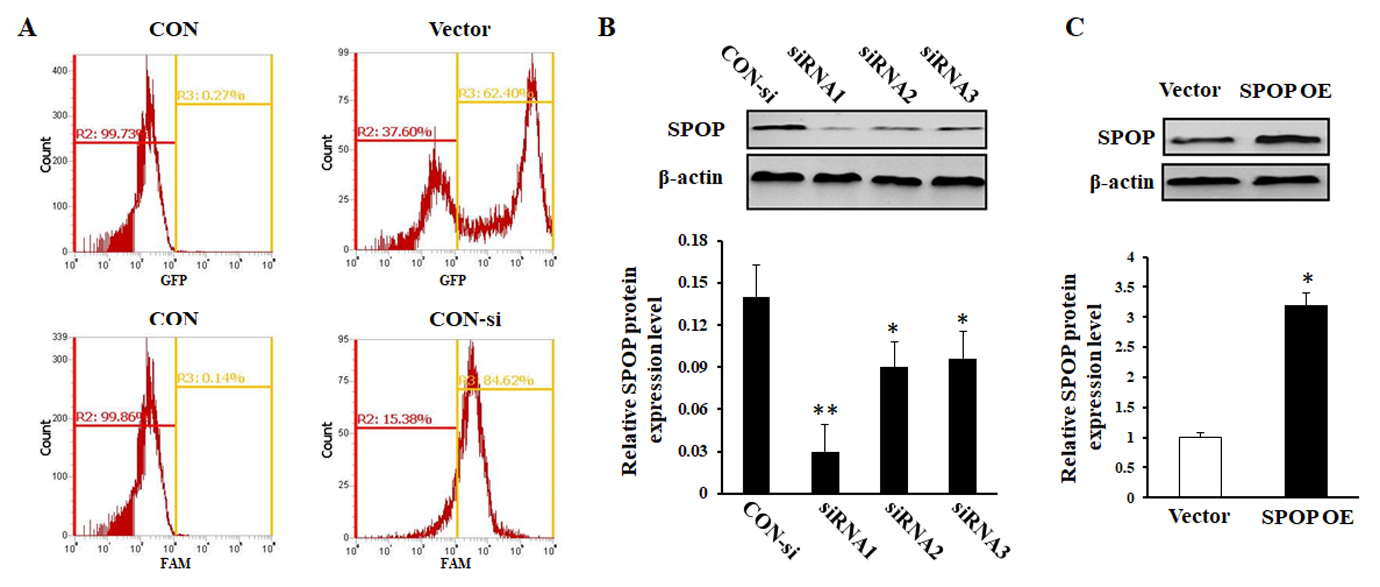


**FIGURE S2 A**. Flow cytometric analysis showed that the efficiency of plasmid transfection is more than 60%, and the efficiency of siRNA transfection is more than 80%. CON represented untransfected cell; Vector represented cells transfected with GFP empty plasmid; CON-si represented FAM-siRNA transfected cells. **B**. SPOP expression was examined by western blot in MKN45 cells transfected with three different siRNAs targeting SPOP (siRNA1, siRNA2, and siRNA3) or CON-si. **C**. Western blot showed that transfection of SPOP overexpression plasmid (SPOP OE) successfully upregulated the protein expression level of SPOP in MKN45 cells. *P < 0.05, **P < 0.001.

**FIGURE S3**


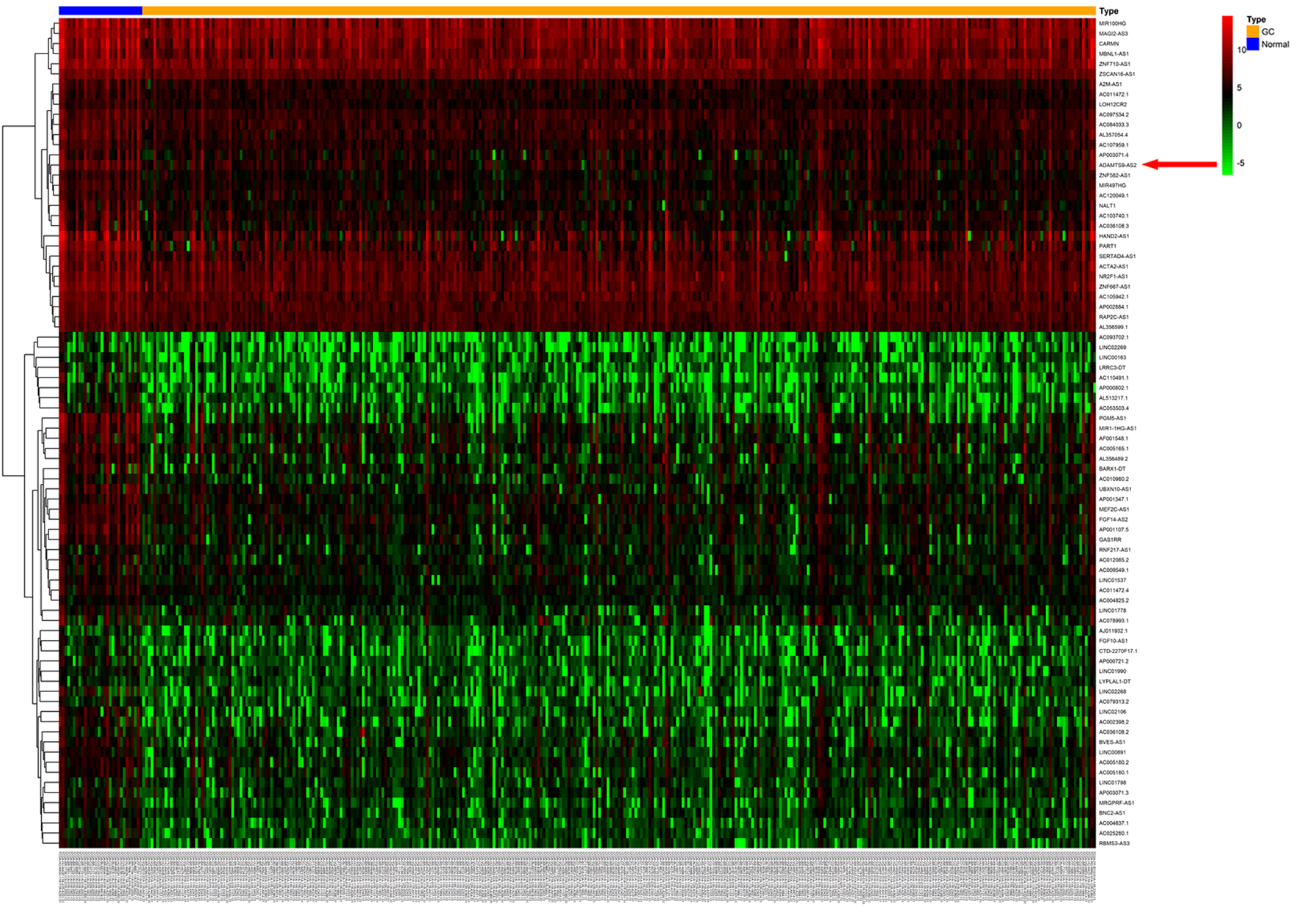


**FIGURE S3** SPOP gene-related lncRNAs were predicted by [bioinformatics](javascript:;) [analysis](javascript:;). The heatmap showed the SPOP gene-related lncRNAs between GC tissues and adjacent normal tissues. These lncRNAs were screened and presented in the rows, and the columns are samples. The green represents down-regulation, while a red represents up-regulation. Red arrow points to ADAMTS9-AS2.

**FIGURE S4**

**
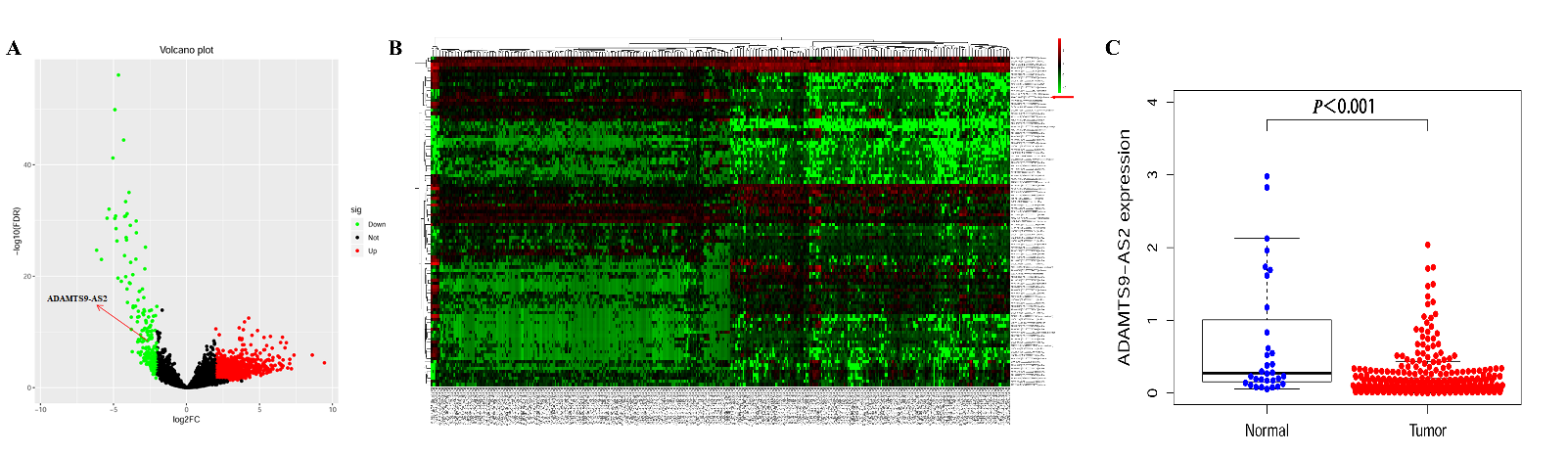
**

**FIGURE S4** The expression levels of ADAMTS9-AS2 were evaluated by [bioinformatics](javascript:;) [analysis](javascript:;). A. Volcano plot of differentially expressed lncRNAs. X-axis: log_2_ (Fold Change); Y-axis: log_10_ (FDR). Red dots: significantly upregulated lncRNAs; Green dots: significantly downregulated lncRNAs; Black dots: non-differentially expressed lncRNAs. B. The heatmap of the top 100 differentially expressed lncRNAs between GC tissues and adjacent normal tissues. The top 100 differentially expressed lncRNAs in GC were screened and presented in the rows, and the columns are samples. The green represents down-regulation, while a red represents up-regulation. The red arrow points to ADAMTS9-AS2. C. The boxplot shows that the mRNA expression levels of ADAMTS9-AS2 were detected in the TCGA database.

**FIGURE S5**

**
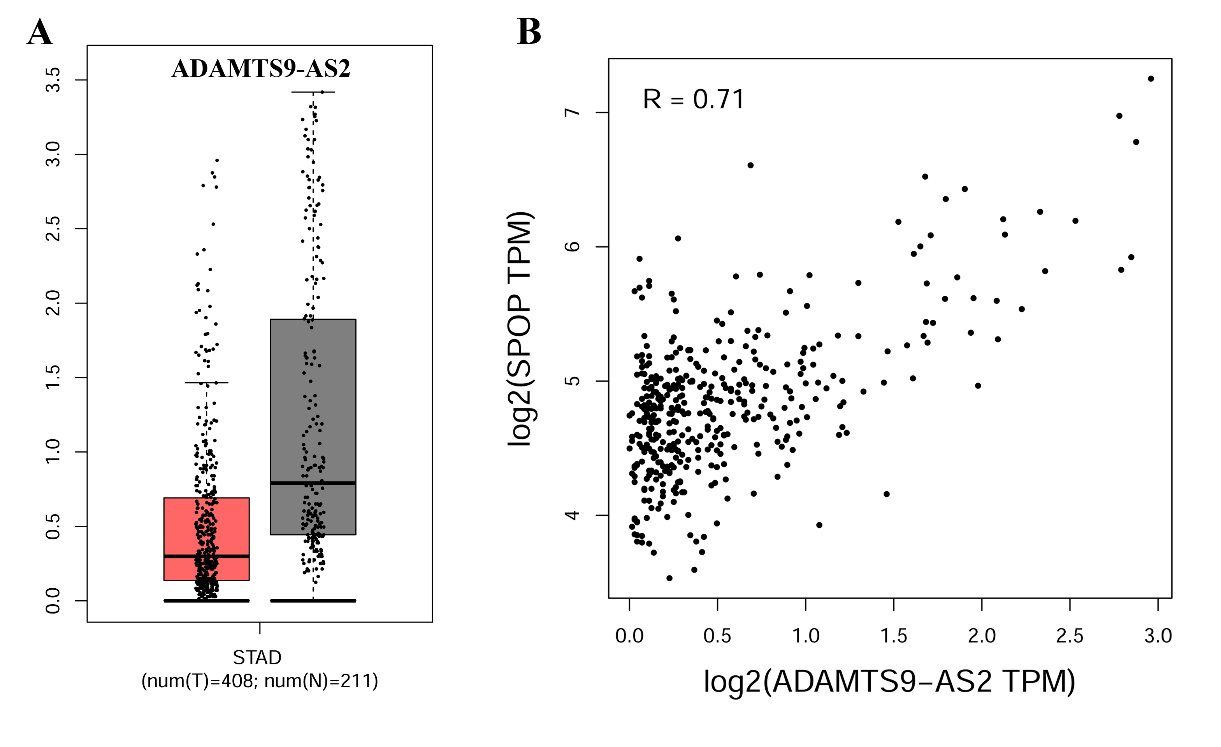
**

**FIGURE S5** GEPIA database verified that ADAMTS9-AS2 was significantly down-regulated in tumor tissues compared with normal tissues (A), and positively correlated with the expression of SPOP in tumor tissues (B) (*P* < 0.001).
